# Condition-dependent effects of phenolic Copigments on anthocyanin stability in Chinese bayberry juice: An HPLC-degradation kinetic study

**DOI:** 10.1016/j.fochx.2026.104368

**Published:** 2026-08-27

**Authors:** Zhengwei Zhang, Xiaodan Chen, Jinning Yin, Luelue Huang

**Affiliations:** School of Food and Drug, Shenzhen Polytechnic University, 7098 Liuxian Avenue, Shenzhen, Guangdong 518055, China

**Keywords:** Anthocyanin stability, Copigmentation, Chinese bayberry juice, Degradation kinetics

## Abstract

This study systematically investigated the condition-dependent effects of six phenolic copigments on the stability of cyanidin-3-O-glucoside (C3G) in model and actual Chinese bayberry juice using spectroscopic, kinetic (HPLC-based), and colorimetric analyses. Flavonoid glycosides, particularly myricitrin, exhibited significant protective effects on C3G in model juice at 90 °C (*P* < 0.05). However, an unexpected reversal was observed: in model juice stored at 37 °C, certain flavonoids and phenolic acids significantly accelerated C3G degradation, reducing its half-life to approximately one-fifth of the control value, whereas the actual juice matrix effectively prevented this reversal. In the presence of ascorbic acid (AA), all copigments mitigated C3G degradation. The protective effect of myricitrin was concentration-dependent, and global nonlinear modeling confirmed significant interactions among copigment type, matrix, temperature, and AA (*P* < 0.001). These findings provide preliminary insights for copigment selection in Chinese bayberry juice processing.

## Highlights

• Certain phenolic acids accelerated C3G degradation in model juice; real juice prevented it.
• HPLC kinetics revealed matrix-, T-, and AA-dependent efficacy of six copigments.
• Myricitrin showed concentration-dependent protection against thermal and oxidative degradation.
• GNLS modeling confirmed significant interactions among copigment type, matrix, T, and AA.

## 1 Introduction

Chinese bayberry (*Myrica rubra* Sieb. et Zucc.) is a fruit native to southern China, valued for its attractive red color, flavor, and nutritional properties. It is rich in anthocyanins, with over 95% present as cyanidin-3-O-glucoside (C3G) (Ye et al., 1994). Although anthocyanins offer various potential health benefits (Gowd et al., 2017; Yang et al., 2023), their chemical instability poses a major challenge for food quality (Ngamwonglumlert et al., 2017).

Intermolecular copigmentation—the non-covalent association between an anthocyanin and a phenolic copigment via π-π stacking, hydrophobic interactions, and hydrogen bonding—is a natural strategy to enhance anthocyanin stability (Trouillas et al., 2016). This interaction produces hyperchromic and bathochromic spectral shifts and can sterically shield the flavylium cation from nucleophilic attack, thereby potentially enhancing resistance to thermal and oxidative stress (Dangles, 2024; J. Wang et al., 2024).

Despite extensive research on phenolic copigments in model systems (Cortez et al., 2017), several gaps remain. Most studies rely on the pH-differential method (Giusti & Wrolstad, 2001), which can be affected by copigmentation itself (J. Lee et al., 2008; S. G. Lee et al., 2016). Others assess stability only at endpoints rather than through full kinetic profiling. Direct comparisons between phenolic acid and flavonoid glycoside copigments under identical conditions are scarce, and few studies integrate kinetic data with colorimetric analysis. Moreover, most research uses simplified buffer systems, overlooking the influence of the complex food matrix on copigment efficacy. Moreover, reported copigment efficacies are sometimes contradictory—e.g., phenolic acids protected at 50–70 °C but accelerated degradation at 90 °C in blackberry wine residue anthocyanins (Fan et al., 2019)—highlighting the need for condition-controlled, matrix-aware investigations.

Based on the structural characteristics of the copigments and prior literature on copigmentation (Fan et al., 2019; Trouillas et al., 2016), we formulated the following hypotheses: (i) flavonoid glycosides (rutin, myricitrin, quercitrin), possessing planar tricyclic cores capable of strong π–π stacking, were expected to exhibit stronger spectral shifts and greater stabilization than phenolic acids (ferulic, gallic, protocatechuic) under most conditions; (ii) the complex matrix of actual Chinese bayberry juice—containing endogenous phenolics, sugars, and organic acids—could modulate copigment efficacy, potentially mitigating adverse effects observed in simple model systems; and (iii) ascorbic acid would accelerate C3G degradation via its pro-oxidant activity, but copigments might partially counteract this effect.

To test these hypotheses, we selected six phenolic copigments representing two major classes: three flavonoid glycosides (rutin, myricitrin, quercitrin) and three phenolic acids (ferulic, gallic, protocatechuic). These compounds are common dietary phenolics with contrasting structural features (extended conjugation vs. smaller aromatic rings) and have been frequently studied individually but rarely compared directly under identical conditions. Using UV–Vis spectroscopy, HPLC-based degradation kinetics, and colorimetry, we systematically investigated their effects on C3G stability in both model and actual Chinese bayberry juice systems under heating (90 °C) and storage (37 °C) conditions, with and without AA. This multifaceted approach aims to provide a quantitative, application-relevant insight into how phenolic copigments stabilize Chinese bayberry anthocyanins, thereby offering a scientific basis for selecting effective color stabilizers in anthocyanin-rich beverages.

## 2 Materials and methods

### 2.1 Chemicals

Cyanidin-3-O-glucoside chloride (C3G, ≥98%, from *Glycine* max(L.) Merr.), protocatechuic acid, gallic acid, ferulic acid, quercitrin, myricitrin, and rutin were obtained from J&K Scientific. Citric acid, ethanol, hydrochloric acid, sodium hydroxide, and formic acid (≥98%, HPLC grade) were purchased from Sinopharm Chemical Reagent Co., Ltd. HPLC-grade methanol and acetonitrile were purchased from Fisher Scientific.

### 2.2 Sample preparation and characterization

#### 2.2.1 Basic characterization of the actual Chinese bayberry juice matrix

The actual Chinese bayberry juice was centrifuged at 12,000 r/min for 10 min at 4 °C, and then characterized for the following parameters.

**pH:** The pH value was determined using a calibrated pH meter (±0.01 pH accuracy). The instrument was calibrated with standard buffer solutions (pH 4.01, 6.86, and 9.18). Measurements were conducted at 25 °C using filtered samples.

**Total Sugar:**Total sugar content was determined using the phenol‑sulfuric acid method. Briefly, 0.5 mL of diluted juice sample was mixed with 1 mL of 5% phenol solution (freshly prepared) in a 25 mL stoppered glass tube. Then, 5 mL of concentrated sulfuric acid was added slowly along the tube wall. After thorough shaking, the mixture was incubated in a 75 °C water bath for 20 min, then cooled to room temperature. The absorbance was measured at 490 nm. Total sugar content was expressed as glucose equivalents (%, *w*/*v*).

**Titratable Acidity (TA):** TA was measured via potentiometric titration. A 5 mL aliquot of juice was titrated with 0.03 mol/L NaOH to an endpoint of pH 8.2. The acidity was calculated using Eq. (1).

(1) $$\mathrm{TA}\left(\%\right)=\frac{C_{\text{NaOH}}\times V_{\text{NaOH}}\times K}{W_{\text{smaple}}}\times 100$$

where *C*_*NaOH*_ is the weight of the sample (g), and *K* is the conversion factor for citric acid (0.064).

**Soluble Solids Content (SSC):** SSC was determined using an Atago PAL-1 digital refractometer (Atago Co., Ltd., Tokyo, Japan) with automatic temperature compensation. Approximately 0.3 mL of sample was placed onto the prism, and the SSC value (%) was read directly. Each sample was measured in triplicate, and the prism was rinsed with distilled water and wiped dry between measurements.

**Total Phenolic Content (TP):** Total phenolic content was measured using the Folin-Ciocalteu method with minor modifications. Briefly, 0.5 mL of sample and 1 mL of Folin-Ciocalteu reagent were added to a 50 mL volumetric flask and mixed vigorously for 30 s. Subsequently, 2 mL of 7.5% sodium carbonate solution was added, and the volume was adjusted to 50 mL with deionized water. After thorough mixing, the mixture was incubated in the dark for 2 h. Absorbance was measured at 760 nm using a UV-2450 spectrophotometer (Shimadzu, Japan). Results were expressed as milligrams of gallic acid equivalents per litre of sample (mg GAE/L).

**Free Individual Phenolic Compounds:** Free individual phenolic compounds were determined by liquid chromatography-mass spectrometry (LC-MS) using a Waters Acquity UPLC system equipped with a photodiode array detector (DAD), electrospray ionization source, and triple quadrupole mass spectrometer (TQD). The chromatographic conditions were as follows: flow rate, 0.3 mL/min; column temperature, 30 °C; mobile phase A, 0.1% formic acid in water; mobile phase B, 0.1% formic acid in acetonitrile. The gradient elution program was: 0 min, 10% B; 0.5 min, 10% B; 15 min, 55% B; 15.5 min, 10% B. The separation was performed on an Acquity BEH C18 reversed-phase column (150 mm × 1.0 mm, 1.7 μm i.d.). The mass spectrometer was operated in negative ion mode with the following parameters: capillary voltage, 3.0 kV; cone voltage, 30 V; source temperature, 100 °C; desolvation temperature, 400 °C; cone gas flow, 50 L/h; desolvation gas flow, 700 L/h; collision energy for full scan, 6 eV and 20 eV. For tandem mass spectrometry (MS/MS) analysis, argon was used as the collision gas at a flow rate of 0.35 mL/min. The optimal cone voltage and collision energy for MS/MS were determined by infusion of standards. Both qualitative and quantitative analyses were performed using external standard calibration.

#### 2.2.2 Preparation of model and actual Chinese bayberry juice

The model Chinese bayberry juice was prepared with a citrate buffer containing 52 mM citric acid. The pH of the model juice was adjusted to 3.3 with 8 M sodium hydroxide solution. C3G stock solution was added to achieve a final concentration of 0.178 mM in 2.7 mL of buffer. Separately, each copigment (protocatechuic acid, gallic acid, ferulic acid, quercitrin, myricitrin, and rutin) was dissolved in ethanol at 8 mM. Then, 0.3 mL of the copigment solution was mixed with the 2.7 mL C3G solution, yielding a total volume of 3 mL with final concentrations of 0.16 mM C3G and 0.8 mM copigment (molar ratio 1:5).  The actual Chinese bayberry juice was prepared as follows. Frozen Chinese bayberries (*Myrica rubra* Sieb. et Zucc. Cv. Dongkui) were first thawed, then juiced. The resulting pulp was filtered through gauze to remove solids. The filtrate was subsequently centrifuged at 4000 r/min for 15 min at 4 °C, and the clear supernatant was collected as the final juice for experiments. The composition of the actual Chinese bayberry juice is provided in **Table S1**.

### 2.3 Effects of six copigments on the spectra of C3G

Using the model Chinese bayberry juice (0.16 mM C3G, 52 mM citric acid, pH adjusted to 3.3 with 6 M NaOH) as the solvent, solutions of six copigments (protocatechuic acid, gallic acid, ferulic acid, quercitrin, myricitrin, and rutin) were prepared at concentrations of 1.6, 0.8, and 0.16 mM, respectively, and mixed thoroughly. The absorbance in the 450–600 nm range was measured using a UV-2600 UV–Vis spectrophotometer (Shimadzu, Japan) with a scanning interval of 0.1 nm. To facilitate the dissolution of the copigments, ethanol was incorporated as a component of the solvent system. The final concentration of ethanol in all treatment groups was maintained at 10% (*v*/v). A control sample, prepared with an equivalent amount of ethanol but devoid of any copigment, was included for comparison. The hyperchromic effect induced by the copigment was expressed as the difference in the maximum absorbance (ΔAmax) between the treatment and control groups, while the bathochromic shift was represented by the difference in the wavelength at maximum absorbance (Δλmax) between the treatment and control groups.

### 2.4 Effects of six copigments on the stability of C3G

For each treatment, a batch of sample solution was prepared and aliquoted into multiple vials. At each designated time point, three vials were sacrificed for analysis (destructive sampling). Solutions of the six copigments (protocatechuic acid, gallic acid, ferulic acid, quercitrin, myricitrin, and rutin) were prepared at a concentration of 8 mM (yielding a molar ratio of C3G to copigment of 1:5) in four different solvent systems: (1) model juice, (2) model juice with 1.6 mM ascorbic acid (AA), (3) actual Chinese bayberry juice, and (4) actual Chinese bayberry juice with 1.6 mM AA. The sample solutions were then thoroughly mixed. A 1.8 mL aliquot of each mixed sample was transferred into a 2 mL amber glass vial. The vials were capped with polypropylene caps equipped with polytetrafluoroethylene (PTFE)-lined septa, which were screwed on finger-tight (i.e., not fully tightened). The sample solutions were then heated in a water bath at 90 °C for 0, 10, 20, 30, 60, and 90 min, respectively. Immediately after each heating interval, the samples were removed, fully tightened and rapidly cooled to room temperature under running tap water.

For the storage group, all samples were first sterilized by heating at 90 °C for 10 min to eliminate microbial contamination, followed by immediate cooling to room temperature. The sterilized samples were then placed in a constant temperature incubator at 37 °C for storage in the dark. They were subsequently removed for analysis at designated time intervals: 0, 2, 4, 6, and 8 days.

### 2.5 Effects of different concentrations of myricitrin on the stability of C3G

Similarly, model and actual Chinese bayberry juice containing myricitrin at concentrations of 0, 0.16, 0.32, 0.8, and 1.6 mM were prepared, corresponding to molar ratios of C3G to myricitrin of 1:0, 1:1, 1:2, 1:5, and 1:10, respectively. These sample sets were then subjected to the identical heating or storage procedures as described in Section 2.4.

### 2.6 Kinetic data analysis

The degradation kinetics of C3G were fitted to a first-order reaction model:

(2) $$C_{t}=C_{0}e^{-\mathrm{kt}}$$

where C_0_ and C_t_ are the C3G concentrations (mg/L) at time zero and time t, respectively, and *k* is the degradation rate constant. The half-life was calculated as *t*_1/2_ = ln2/*k*.

### 2.7 Quantification of C3G

C3G was quantified by HPLC according to Z. Zhang, Li, et al. (2020) with modifications. Samples were centrifuged at 12,000 r/min for 10 min at 4 °C. The supernatant was filtered through a 0.22 μm aqueous membrane filter prior to HPLC analysis. C3G was quantified using an Agilent 1260 Infinity II liquid chromatograph (Agilent Technologies, Santa Clara, CA, USA) equipped with a diode array detector (DAD; G4212B, Agilent Technologies) and operated by Agilent OpenLAB CDS software. Separation was performed on a Waters XBridge BEH C18 column (5 μm, 250 mm × 4.6 mm i.d.; Waters, Milford, USA). The injection volume was 20 μL, the flow rate was 0.6 mL/min, and the column oven was maintained at 30 °C. The mobile phase consisted of (A) water/formic acid (100:1, *v*/v) and (B) acetonitrile/formic acid (100,1, *v*/v), with the following gradient: 0–12 min, 5% → 50% B; 12–14 min, 50% → 100% B; 14–15 min, 100% → 5% B; followed by re-equilibration for 5 min. Chromatograms were recorded at 280 and 510 nm. Quantification was performed using a single-point external standard method. Against a known concentration of C3G standard (0.16 mM). The concentration of C3G in the samples was calculated based on the ratio of peak areas.

### 2.8 Color analysis of juice

CIELab parameters were determined by a ST-700d plus spectrophotometer (3nh, China). The simulated color was generated based on the ARGB hex color code by SQCX software (version 1.9.11.111, 3nh, China). After centrifugation at 12,000 r/min for 10 min at 4 °C, 0.8 mL of the clear supernatant was placed in a white polytetrafluoroethylene (PTFE) evaporation dish (20 mm in diameter, 5 mm in depth) for color documentation. A ring-shaped colorimetric card, printed with color patches of known *L, a, b* values, was positioned around the dish. The assembly was then placed inside a constant-brightness light box. Photographs were captured using the same iPhone 16 Pro smartphone, mounted at a fixed height above the sample.

### 2.9 Statistical analysis

Statistical analyses were performed using GraphPad Prism 11.0 (Dotmatics, Boston, MA). The degradation rate constant *k* and the half-life *t*_1/2_ were obtained directly from the nonlinear regression output (one-phase decay). Due to the destructive sampling design (three vials sacrificed at each time point), each treatment yielded a single *k* value from the pooled mean of triplicate bottles. To compare each treatment with the control, we used the extra sum-of-squares F test, which evaluates whether the best-fit value of *k* differs significantly between two datasets. Comparisons were performed and the resulting *P* values were corrected using the Holm-Bonferroni method. A corrected *P* < 0.05 was considered statistically significant. The standard errors (SE) of *k* and *t*_1/2_ were also obtained directly from the nonlinear regression output. For kinetic data, error bars for the raw *Ct* data points in figures represent the standard deviation (SD) of three vials per time point (destructive sampling). All data figures were generated using OriginPro 2021 (OriginLab Corporation, Northampton, MA, USA).

Global nonlinear least squares (GNLS) analysis was performed using the gnls function in the nlme package in R (version 4.x, R Foundation for Statistical Computing, Vienna, Austria). No random effects were included due to the destructive sampling design. The model was based on first-order degradation kinetics, with log-transformed degradation rate constants (ln*k*) modeled as a linear function of fixed effects, including copigment treatment (type and concentration), matrix, temperature, ascorbic acid (AA), and their interactions. Separate residual variances were estimated for each of the eight experimental conditions (two matrices × two temperatures × ± AA) to account for heteroscedasticity. Model fit was evaluated using pseudo-R^2^ and standardized residual diagnostics. ANOVA tables were generated for the full-sample model and two sub-models (copigment type at fixed 1:5 ratio, and myricitrin concentration gradient). Post-hoc comparisons between each treatment and its respective control were performed using Dunnett's test, with *P* values adjusted for multiple comparisons.

For color parameters, measurements were taken at two time points (initial and final storage). At each time point, one-way ANOVA (SPSS software, Version 20, IBM, USA) was performed to test differences among treatments, followed by Tukey's HSD post-hoc test  for all pairwise comparisons. The significance level was set at 0.05. Pearson correlation analysis was performed to evaluate the relationship between C3G retention and color parameters (*ΔE* and a*). C3G retention was calculated as C_final / C_initial for each treatment. Correlations were analyzed separately for two representative conditions (model juice, 90 °C, no AA and model juice, 37 °C, no AA). The correlation coefficient (*r*) and its statistical significance (*P* value) were computed using SPSS software (Version 20, IBM, USA).

## 3 Results and discussion

### 3.1 Effects of six copigments on the spectra of C3G

**Fig. 1:**
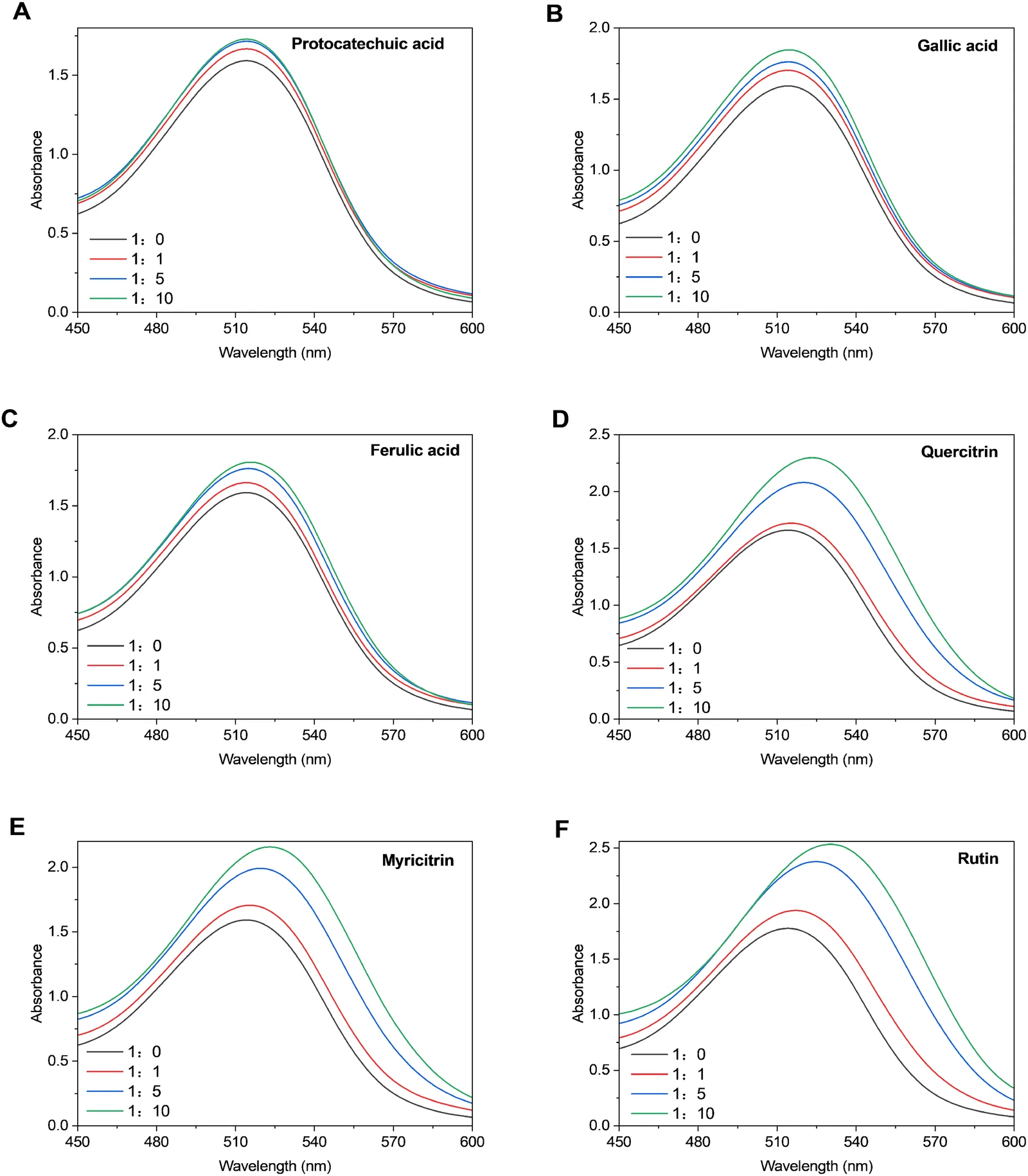
Absorbance spectra of model Chinese bayberry juice (0.16 mM cyanidin-3-O-glucoside, 52 mM citric acid, pH 3.3) with addition of protocatechuic acid (A), gallic acid (B), ferulic acid (C), quercitrin (D), myricitrin (E), and rutin (F) at various molecular ratios with cyanidin-3-glucoside (cyanidin-3-glucoside / copigment).

To investigate the interactions between different phenolic compounds and cyanidin-3-O-glucoside (C3G) at the molecular level, the effects of six copigments at three different molar ratios on the UV–Vis absorption spectra of C3G solutions were determined. As shown in Fig. 1, all tested copigments induced spectral changes to varying degrees, confirming the occurrence of intermolecular copigmentation, although their efficacy differed significantly depending on the type and concentration.

A hyperchromic effect was universally observed and exhibited a clear concentration dependence and similar phenomenon was also reported (Z. Xu et al., 2022). The addition of all copigments increased the absorbance (A_max_) of C3G at its maximum absorption wavelength (λ_max_ ≈ 514 nm). The flavonoid glycosides (rutin, myricitrin, and quercitrin) induced the most pronounced hyperchromic effect. At the highest addition ratio (1:10), the increases in absorbance (ΔA_max_) for rutin, myricitrin, and quercitrin reached 0.76, 0.57, and 0.64, respectively. In contrast, the phenolic acids (protocatechuic acid, gallic acid, and ferulic acid) showed much weaker effects, with their respective ΔA_max_ values at the same ratio being only 0.14, 0.25, and 0.21. The hyperchromic effect of ferulic acid is largely consistent with the findings reported by Kanha et al. (2019). In their system with an anthocyanin-to-ferulic acid molar ratio of 1:10, a hyperchromic effect of 11.1% was observed, while in our experiment, the corresponding value was 13.8%, possibly due to differences in anthocyanin structure or matrix composition. In another study investigating the copigmentation of anthocyanins in black chokeberry, the researchers found that the hyperchromic effects induced by gallic acid and protocatechuic acid were comparable, with neither consistently stronger than the other (Klisurova et al., 2019). This differs from our observation that gallic acid induced a stronger hyperchromic effect than protocatechuic acid. This discrepancy may be attributable to differences in the specific anthocyanin species involved.

A bathochromic shift demonstrated even more distinct structural differences. The red shift (Δλ_max_) is a key indicator of strong π-π stacking interactions between the copigment and the C3G chromophore. As summarized in Table 1, the three flavonoid glycosides all induced intense and concentration-dependent bathochromic shifts. Rutin was the most effective, producing shifts of 2.8, 10.6, and 16.2 nm at ratios of 1:1, 1:5, and 1:10, respectively. Myricitrin and quercitrin also caused significant shifts, reaching Δλ_max_ values of 9.0 and 9.1 nm at the 1:10 ratio. However, the bathochromic shifts induced by the three phenolic acids were extremely limited. Even at the 1:10 ratio, ferulic acid produced only a 1.7 nm shift, while the shifts for gallic acid and protocatechuic acid did not exceed 0.4 nm. Consistent with the findings of this study, the research by Ertan et al. (2019) also observed that the bathochromic shift induced by gallic acid was very weak. For protocatechuic acid, no measurable shift (Δλ_max_ = 0) was detected even at higher concentrations.

**Table 1:** Copigment-induced hyperchromic effect and bathochromic shift of the spectra of cyanidin-3-O-glucoside (C3G). The control group contained no copigment.

Copigment	Copigment: Anthocyanin Molar Ratio	A_max_	ΔA_max_	λ_max_	Δλ_max_
Protocatechuic acid	Control	1.59	0	514.1	0
	1	1.67	0.08	514.2	0.1
	5	1.72	0.12	514.1	0
	10	1.73	0.14	514.1	0

Gallic acid	Control	1.59	0	514.1	0
	1	1.70	0.11	514.2	0.1
	5	1.76	0.17	514.4	0.3
	10	1.85	0.25	514.5	0.4

Ferulic acid	Control	1.59	0	514.1	0
	1	1.66	0.07	514.2	0.1
	5	1.76	0.17	514.9	0.8
	10	1.81	0.21	515.8	1.7

Quercitrin	Control	1.66	0	514.2	0
	1	1.72	0.06	515.4	1.2
	5	2.08	0.42	520.1	5.9
	10	2.30	0.64	523.3	9.1

Myricitrin	Control	1.59	0	514.1	0
	1	1.71	0.11	515.4	1.3
	5	1.99	0.40	519.5	5.4
	10	2.16	0.57	523.1	9

Rutin	Control	1.78	0	514.2	0
	1	1.94	0.16	517.0	2.8
	5	2.38	0.60	524.8	10.6
	10	2.53	0.76	530.4	16.2

These spectral data provide direct evidence for intermolecular copigmentation. The significant bathochromic shifts are consistent with face-to-face π–π stacking interactions, which may account for the high efficacy of flavonoid glycosides as copigments (Trouillas et al., 2016). Their planar structures can overlap with the pyrylium ring of C3G, lowering excitation energy, whereas phenolic acids engage in weaker π–π stacking due to their smaller size. Among the flavonoid glycosides, rutin produced the strongest hyperchromic and bathochromic effects, plausibly related to its disaccharide rutinose moiety, which confers a larger molecular volume (375.2 vs. 175.6 Å^3^ for quercetin; Fan et al. (2019)) and is described as “one of the mainly completely planar polyphenols, rendering complete overlapping of π–π stacking” (Fan et al., 2019). The bulkier size may inhibit rotational freedom, stabilizing the flavylium cation (Chen et al., 2022; Fan et al., 2019). These findings suggest that flavonoid glycosides with stronger π–π interactions may form more stable copigmentation complexes, potentially offering enhanced protection to C3G. Efficient copigments are known to slow anthocyanin degradation by limiting chalcone formation (Trouillas et al., 2016), and rutin has been shown to produce strong spectral shifts, reduce degradation rates (Chen et al., 2022) and stabilize color over prolonged storage (Hernández-Herrero & Frutos, 2015).

### 3.2 Effects of six copigments on C3G stability

**Fig. 2:**
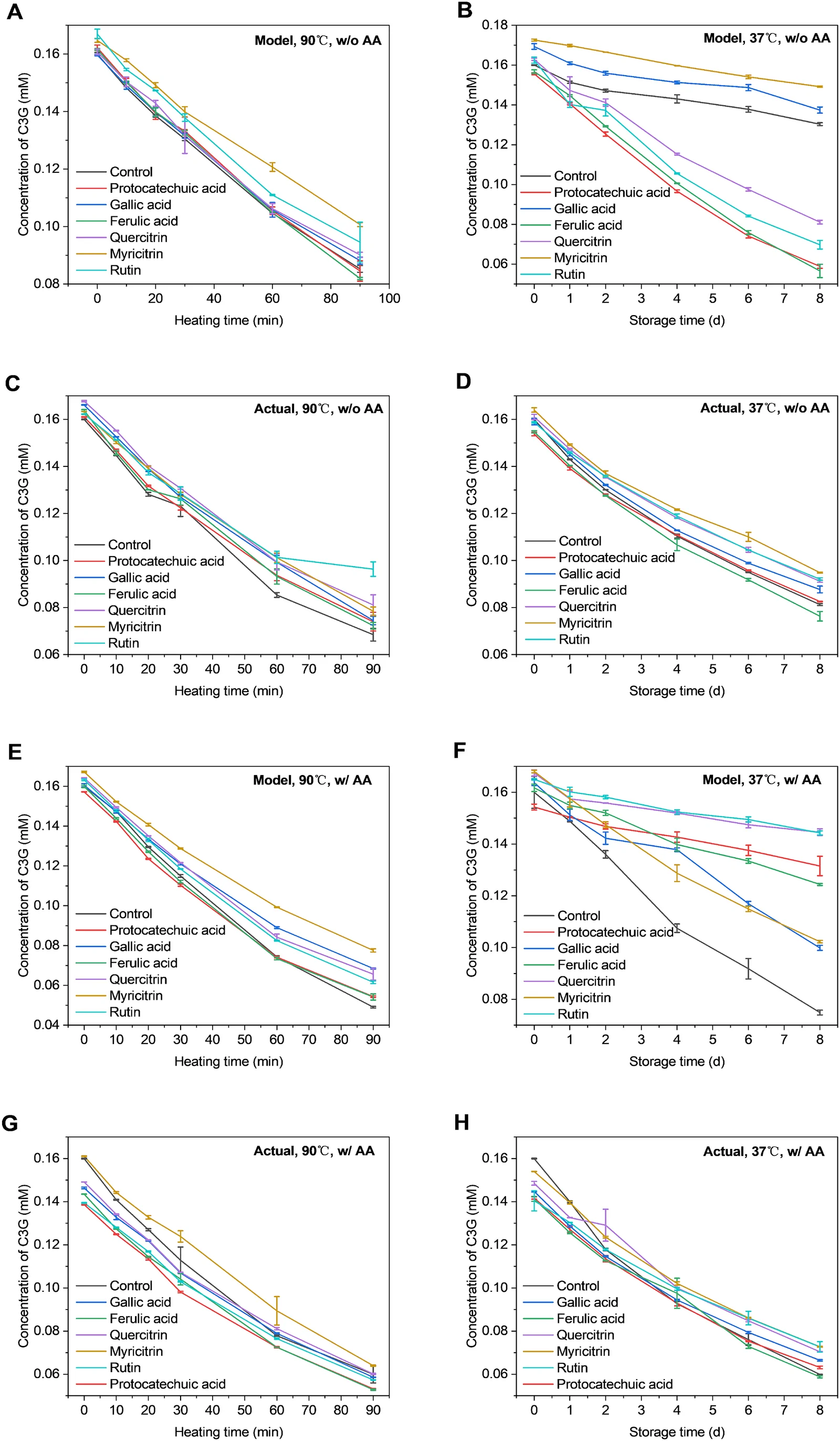
Degradation profiles of cyanidin-3-glucoside (C3G) as influenced by six phenolic copigments under varying juice matrix, temperature, and ascorbic acid (AA) addition. Panels are arranged by two temperatures (90 °C, 37 °C), two juice matrices (model, actual), and with or without 1.6 mM AA. The C3G-to-copigment molar ratio was fixed at 1:5 in all panels.

To validate the spectroscopic predictions and assess practical application potential, we systematically evaluated the effects of six copigments on the degradation kinetics of C3G under varying conditions: model vs. actual Chinese bayberry juice, heating at 90 °C vs. storage at 37 °C, and with or without 1.6 mM ascorbic acid (AA) (Fig. 2**,** Table 2). Of note, the actual Chinese bayberry juice inherently contained ascorbic acid at ∼21 mg/100 mL(Yu et al., 2013), a level close to the 1.6 mM AA (∼28 mg/100 mL) added in the model systems and within the typical fruit range of 11.9–114.6 mg/100 g FW summarized by S. Zhang et al. (2022). This endogenous AA may contribute to the matrix-specific behaviors discussed below. The results confirmed that the effects of copigments are highly condition-dependent. Their performance in stabilizing C3G partly corresponded to, but was also complexly modulated by, the environmental conditions beyond their spectral characteristics.

**Table 2:** Degradation kinetic parameters of cyanidin-3-O-glucoside (C3G) with six different copigments under varying conditions of matrix, temperature, and ascorbic acid (AA) addition.

Without AA							With 1.6 mM AA						
Matrix	T (°C)	Copigment	*R*^2^	*k*	*t*_1/2_	P (*k*)	Matrix	T (°C)	Copigment	*R*^2^	*k*	*t*_1/2_	P (*k*)
Model juice	90	Control	0.984	0.0069 ± 0.003	100.3 ± 3.8 min		Model juice	90	Control	0.994	0.0129 ± 0.0003	53.9 ± 1.3 min	
		Protocatechuic acid	0.985	0.0071 ± 0.003	97.4 ± 3.6 min	ns			Protocatechuic acid	0.964	0.0073 ± 0.0004	95.4 ± 5.5 min	ns
		Gallic acid	0.982	0.0066 ± 0.003	105.8 ± 4.4 min	ns			Gallic acid	0.944	0.0071 ± 0.0005	97.2 ± 7.0 min	ns
		Ferulic acid	0.987	0.0073 ± 0.003	94.5 ± 3.2 min	ns			Ferulic acid	0.963	0.0083 ± 0.0005	83.2 ± 4.9 min	ns
		Quercitrin	0.964	0.0065 ± 0.003	106.5 ± 6.0 min	ns			Quercitrin	0.956	0.0066 ± 0.0004	104.7 ± 6.6 min	*
		Myricitrin	0.972	0.0053 ± 0.003	131.1 ± 6.4 min	***			Myricitrin	1.000	0.0086 ± 0.0001	80.9 ± 0.5 min	***
		Rutin	0.973	0.0064 ± 0.003	109.1 ± 5.3 min	ns			Rutin	0.962	0.0064 ± 0.0004	109.0 ± 6.3 min	**
	37	Control	0.951	0.0231 ± 0.0014	30.0 ± 1.8 d			37	Control	0.989	0.0961 ± 0.0029	7.2 ± 0.2 d	***
		Protocatechuic acid	0.998	0.1226 ± 0.0016	5.7 ± 0.1 d	***			Protocatechuic acid	0.950	0.0191 ± 0.0012	36.3 ± 2.2 d	***
		Gallic acid	0.931	0.0226 ± 0.0017	30.6 ± 2.2 d	ns			Gallic acid	0.962	0.0562 ± 0.0031	12.3 ± 0.7 d	***
		Ferulic acid	0.992	0.1234 ± 0.0033	5.6 ± 0.2 d	***			Ferulic acid	0.986	0.0324 ± 0.0010	21.4 ± 0.7 d	***
		Quercitrin	0.989	0.0860 ± 0.0026	8.1 ± 0.2 d	***			Quercitrin	0.871	0.0164 ± 0.0017	42.2 ± 4.3 d	***
		Myricitrin	0.996	0.0187 ± 0.0003	37.1 ± 0.6 d	**			Myricitrin	0.994	0.0629 ± 0.0014	11.0 ± 0.2 d	***
		Rutin	0.987	0.1057 ± 0.0035	6.6 ± 0.2 d	***			Rutin	0.970	0.0159 ± 0.0007	43.5 ± 2.0 d	***

Actual juice	90	Control	0.990	0.0097 ± 0.0003	71.2 ± 2.2 min		Actual juice	90	Control	0.993	0.0113 ± 0.0003	61.1 ± 1.7 min	
		Protocatechuic acid	0.995	0.0088 ± 0.0002	79.0 ± 1.6 min	**			Protocatechuic acid	0.964	0.0073 ± 0.0004	95.4 ± 5.5 min	ns
		Gallic acid	0.998	0.0088 ± 0.0001	78.7 ± 1.1 min	**			Gallic acid	0.944	0.0071 ± 0.0005	97.2 ± 7.0 min	ns
		Ferulic acid	0.991	0.0090 ± 0.0003	76.7 ± 2.3 min	ns			Ferulic acid	0.963	0.0083 ± 0.0005	83.2 ± 4.9 min	ns
		Quercitrin	0.994	0.0083 ± 0.0002	83.2 ± 1.9 min	***			Quercitrin	0.956	0.0066 ± 0.0004	104.7 ± 6.6 min	ns
		Myricitrin	0.999	0.0080 ± 0.0001	86.3 ± 1.0 min	***			Myricitrin	0.991	0.0099 ± 0.0003	69.9 ± 2.0 min	**
		Rutin	0.960	0.0065 ± 0.0004	107.4 ± 6.4 min	***			Rutin	0.962	0.0064 ± 0.0004	109.0 ± 6.3 min	**
	37	Control	0.995	0.0850 ± 0.0017	8.2 ± 0.2 d			37	Control	0.993	0.1258 ± 0.0032	5.5 ± 0.1 d	
		Protocatechuic acid	0.997	0.0772 ± 0.0013	9.0 ± 0.2 d	***			Protocatechuic acid	0.998	0.1030 ± 0.0013	6.7 ± 0.1 d	***
		Gallic acid	0.993	0.0764 ± 0.0019	9.1 ± 0.2 d	**			Gallic acid	0.996	0.0988 ± 0.0018	7.0 ± 0.1 d	***
		Ferulic acid	0.997	0.0880 ± 0.0014	7.9 ± 0.1 d	ns			Ferulic acid	0.986	0.1062 ± 0.0038	6.5 ± 0.2 d	***
		Quercitrin	0.995	0.0709 ± 0.0014	9.8 ± 0.2 d	***			Quercitrin	0.980	0.0936 ± 0.0041	7.4 ± 0.3 d	***
		Myricitrin	0.989	0.0665 ± 0.0020	10.4 ± 0.3 d	***			Myricitrin	0.997	0.0960 ± 0.0016	7.2 ± 0.1 d	***
		Rutin	0.998	0.0681 ± 0.0009	10.2 ± 0.1 d	***			Rutin	0.991	0.0829 ± 0.0022	8.4 ± 0.2 d	***

Values are mean ± SE. The rate constant *k, t*_1/2_ and their SE were obtained from nonlinear regression (first-order model, GraphPad Prism 11.0, extra sum-of-squares F test). Significance of difference in *k* between each treatment and the control was assessed by the extra sum-of-squares F test with Holm-Bonferroni correction; ns, not significant (*P* ≥ 0.05); * (*P* < 0.05); ** (*P* < 0.01); *** (*P* < 0.001). The molar ratio of C3G to all copigments was 1:5.

An intriguing observation that further corroborates the occurrence of intermolecular interactions was the discrepancy in the initial C3G content measured by HPLC. Despite being prepared at the same nominal concentration (0.16 mM), the measured C3G concentration at time zero varied between the copigment-containing groups and the control. This phenomenon is highly likely a direct manifestation of copigmentation: the formation of non-covalent complexes between C3G and the copigments could alter the chromatographic behavior or detector response of C3G, introducing a systematic quantification bias. Importantly, the magnitude and direction of this initial discrepancy itself provided additional evidence for the varying strength of interaction among different copigment classes, consistent with the spectroscopic data in Section 3.1.

To evaluate how these verified intermolecular interactions translated into macroscopic stability under practical conditions, we first examined systems without added AA. The effects of copigments on C3G stability were largely governed by temperature and matrix. Under 90 °C heating, most copigments exhibited protective effects, with flavonoid glycosides generally outperforming phenolic acids. This aligns with the trend predicted by their stronger π-π stacking capability, even though phenolic acids at the same molar concentration can dissociate more protons, leading to a lower system pH—a condition generally known to favor anthocyanin stability, including that of C3G. In the model juice (Table 2), myricitrin showed the most prominent protection, significantly extending the half-life (t_1/2_) of C3G from 99.0 min in the control to 126.0 min (degradation rate constant *k* decreased from 0.0070 to 0.0055 min^−1^). Rutin and quercitrin also performed well. In contrast, phenolic acids showed weaker effects. In the actual Chinese bayberry juice, rutin demonstrated the strongest protective effect, substantially extending the t_1/2_ from 72.2 min to 113.6 min. An interesting phenomenon was that ferulic acid and protocatechuic acid, which showed weaker protection in the model juice, exhibited a more pronounced protective trend in the actual juice. This suggests that certain components in the real matrix (e.g., other phenolic compounds, proteins, or polysaccharides) may have synergistic effects with them (Chen et al., 2022; Fernandes et al., 2020), highlighting the importance of matrix composition.

Under 37 °C storage conditions, a key “condition-dependent reversal” occurred in the model system. During storage in the model juice, the gallic acid group showed little difference from the control, which is consistent with the findings in model and actual blueberry juice where no significant difference was found in C3G between the addition of gallic acid and the control group during storage (Cao et al., 2023; L. L. Zhang, Wang, et al., 2020). However, the degradation rate constants (k) for the protocatechuic acid, rutin, quercitrin, and ferulic acid treatment groups were abnormally high (>0.1 days^−1^), resulting in much shorter t_1/2_ values (5.4–6.5 days) compared to the control (30.3 days). This result suggests that, under this specific condition (model juice, 37 °C, without AA), certain copigments unexpectedly accelerated C3G degradation. The mechanism underlying this effect remains unclear and warrants further investigation. Similarly, a comparable reversal phenomenon that ferulic acid and protocatechuic acid could slow anthocyanin degradation was observed (Cao et al., 2023; Fan et al., 2019). But at 90 °C, phenolic acids including ferulic and protocatechuic acids accelerated anthocyanin degradation (Fan et al., 2019). A difference, however, is that in their study, flavonoids like rutin slowed anthocyanin degradation at all tested temperatures (50, 70, and 90 °C). This discrepancy might be attributed to the use of a different matrix (a buffer solution containing blackberry wine residue anthocyanins).

Therefore, the mechanism responsible for this condition-dependent reversal—whether involving oxidative pathways or other processes—remains to be elucidated. In contrast to the heating results, during storage in the actual juice, all copigments exhibited clear protective effects. This clearly demonstrates that the complexity of the real food matrix can effectively maintain the chemical stability of the copigments, ensuring they exert positive protective functions and thereby avoiding the “reversal” phenomenon. Other antioxidant components or more complex interaction networks present in the real matrix may inhibit the degradation or harmful side reactions of the copigments themselves (Cavalcanti et al., 2011). It is worth noting that in the model juice without AA, myricitrin extended the C3G half-life from 99 min to 126 min during 90 °C heating and from 30.0 days to 37.1 days during 37 °C storage. This aligns with the findings of H. Xu et al. (2015), who reported that myricitrin extended the half-life of grape skin anthocyanins from 495 min to 693 min during 90 °C heating.

The addition of AA altered the redox environment, becoming a dominant factor. In the strong oxidative stress of 90 °C heating, AA itself, acting as a potent pro-oxidant, drastically accelerated C3G degradation. This is consistent with findings in published studies (Stebbins et al., 2016; Z. Zhang, et al., 2022). Under 37 °C storage conditions with added AA, all copigments demonstrated protective effects. For instance, quercitrin and rutin extended the C3G half-life in the model juice from 7.2 days (control) to 42.2 days and 43.5 days, respectively. The protective effect of quercitrin on anthocyanins is rarely reported, while that of rutin has been documented in several studies (da Silva et al., 2025; Hernández-Herrero and Frutos, 2015). A key change, however, was the relative prominence of the protective effect of phenolic acids, especially protocatechuic acid. In the model juice, protocatechuic acid was the most effective, even outperforming some flavonoid glycosides (e.g., myricitrin). The relative enhancement of protocatechuic acid's protection in the presence of AA may be related to its specific antioxidant mechanism, warranting further investigation. Gallic acid extended the C3G half-life in the model juice from 53.9 min to 97.2 min at 90 °C and from 7.2 days to 12.0 days at 37 °C. This is largely consistent with the findings of Lin et al. (2023), who reported that gallic acid increased the half-life of anthocyanins in a model beverage from 3.33 days to 14.87 days at 25 °C. This is likely because under intense thermal-oxidative conditions, the antioxidant capacity of different phenolic compounds to scavenge reactive oxygen species (ROS) generated by AA begins to play a significant role (Kostka et al., 2020), jointly determining the final protective outcome along with their copigmentation ability. Studies have shown that in the presence of AA, certain phenolic acids may protect anthocyanins indirectly by competitively scavenging free radicals or interacting with AA/its oxidation products (Roidoung et al., 2016). Similarly, regarding the ability to resist anthocyanin degradation caused by hydrogen peroxide, one study found that rutin was more effective than ferulic acid, which in turn was more effective than protocatechuic acid (Fan et al., 2019). The finding that rutin is more effective than ferulic acid and protocatechuic acid is consistent with our study. However, the order of effectiveness between ferulic acid and protocatechuic acid was opposite to ours, indicating differences between oxidation degradation caused by ascorbic acid and hydrogen peroxide.

These results reveal that the influence of copigments on C3G stability is a complex process dynamically regulated by multiple factors: the copigment's own structure, the food matrix, temperature, and the redox environment. Flavonoid glycosides, due to their strong π-π stacking ability, generally outperformed phenolic acids under most conditions (especially in heat treatment and in real systems without AA), consistent with the spectroscopic results in Section 3.1. However, under the specific condition of “model juice, 37°C storage, without AA,” they produced negative effects due to their own chemical instability, indicating the potential limitations of model systems in predicting long-term storage behavior. In contrast, the real juice matrix supported the function of the copigments. Furthermore, the addition of AA introduced oxidative stress, shifting the relative importance of different phenolic compounds. The relative enhancement of the protective effect of phenolic acids under thermal-oxidative conditions suggests that in food systems rich in ascorbic acid, selecting copigments requires balancing both their antioxidant capacity and their copigmentation ability.

It is noteworthy that the accelerated degradation of C3G by certain copigments under the specific condition of model juice, 37 °C, without AA represents one of the most unexpected findings of this study. However, the present study did not measure reactive oxygen species (ROS), dissolved oxygen, copigment degradation products, or transition metal ion concentrations. Therefore, whether this effect involves a true pro-oxidant mechanism or other pathways cannot be conclusively determined. Elucidating the molecular basis of this condition-dependent reversal will require dedicated oxidative chemistry studies in future work.

### 3.3 Effect of myricitrin concentration on C3G stability

**Fig. 3:**
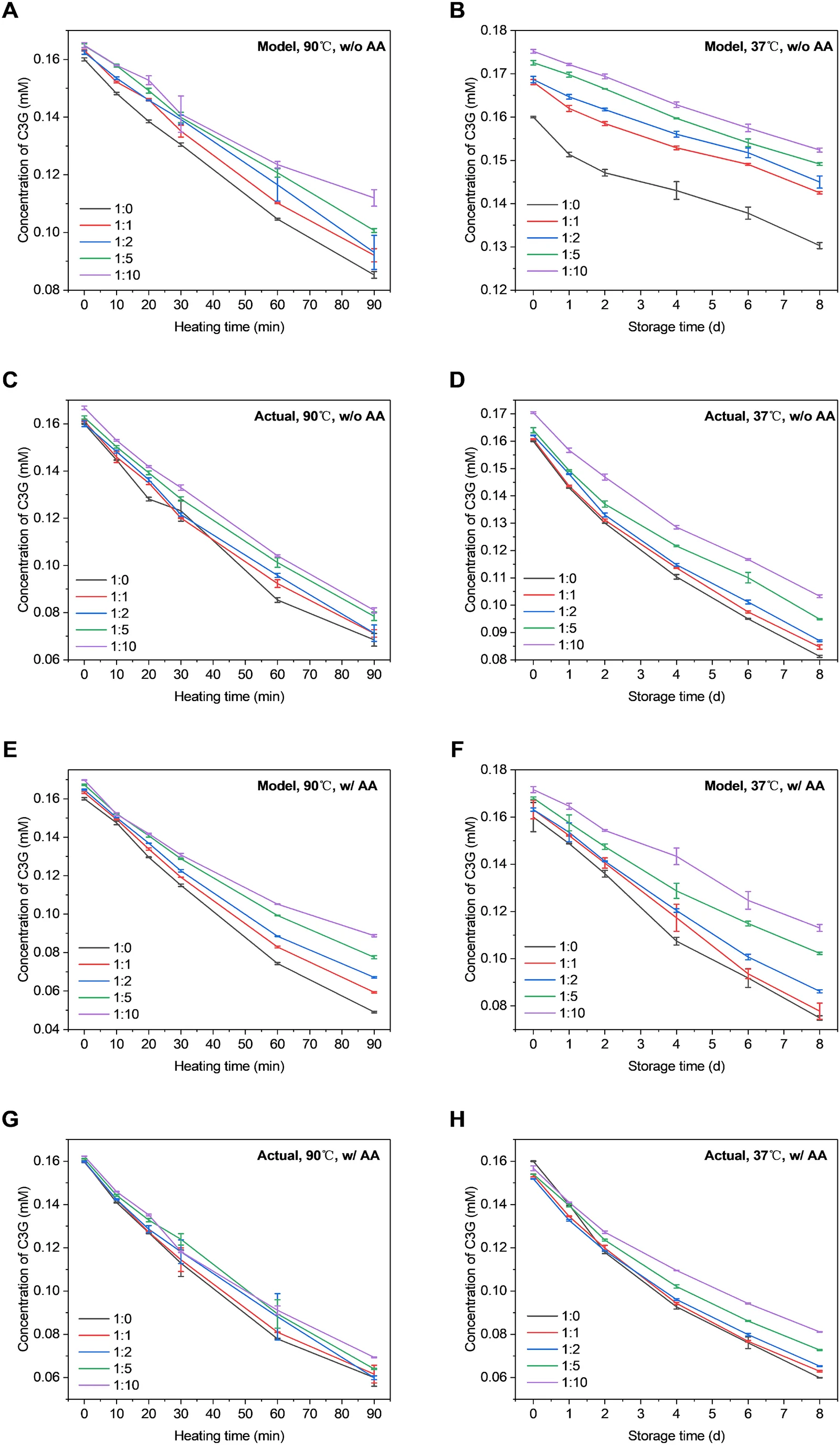
Degradation profiles of cyanidin-3-glucoside (C3G) in model and actual Chinese bayberry juices as influenced by myricitrin concentration, temperature, and ascorbic acid (AA) addition. Panels are arranged as two temperatures (90 °C, 37 °C), two juice matrices (model, actual), and with or without 1.6 mM AA, with C3G-to-myricitrin molar ratios of 1:0, 1:1, 1:2, 1:5, and 1:10 within each panel.

Among the three flavonoid glycosides screened, myricitrin was selected for the concentration-dependent study because it provided the most consistent protection across all tested conditions (see Table 2) and is naturally present in Chinese bayberry (X. Zhang et al. (2015), **Table S1**), making it directly relevant to the juice matrix. We therefore investigated the effect of its addition concentration (C3G:myricitrin molar ratios ranging from 1:0 to 1:10) (Fig. 3, Table 3). The results showed a clear concentration dependence of its protective effect, providing stronger evidence that myricitrin can protect C3G under most tested conditions, except in model juice at 37 °C without ascorbic acid where no significant effect was observed.

**Table 3:** Degradation kinetic parameters of cyanidin-3-O-glucoside (C3G) with myricitrin under varying conditions of matrix, temperature, and ascorbic acid (AA) addition.

Without AA							With 1.6 mM AA						
Matrix	T (°C)	Molar ratio (C3G:myricitrin)	*R*^2^	*k*	*t*_1/2_	P (*k*)	Matrix	T (°C)	Molar ratio (C3G:myricitrin)	*R*^2^	*k*	*t*_1/2_	P (*k*)
Model juice	90	1:0	0.984	0.0069 ± 0.0003	100.3 ± 3.8 min		Model juice	90	1:0	0.994	0.0129 ± 0.0003	53.9 ± 1.3 min	
		1:1	0.981	0.0063 ± 0.0003	109.8 ± 4.4 min	ns			1:1	0.998	0.0113 ± 0.0002	61.5 ± 0.8 min	***
		1:2	0.971	0.0059 ± 0.0003	117.5 ± 5.9 min	*			1:2	0.999	0.0102 ± 0.0001	68.3 ± 0.7 min	***
		1:5	0.972	0.0053 ± 0.0003	131.1 ± 6.4 min	***			1:5	1.000	0.0086 ± 0.0001	80.9 ± 0.5 min	***
		1:10	0.883	0.0049 ± 0.0003	142.7 ± 15.3 min	***			1:10	0.991	0.0073 ± 0.0002	94.3 ± 2.6 min	***
	37	1:0	0.951	0.0231 ± 0.0014	30.0 ± 1.8 d			37	1:0	0.989	0.0961 ± 0.0029	7.2 ± 0.2 d	
		1:1	0.978	0.0193 ± 0.0008	35.9 ± 1.4 d	*			1:1	0.987	0.0924 ± 0.0030	7.5 ± 0.2 d	ns
		1:2	0.987	0.0181 ± 0.0006	38.2 ± 1.2 d	**			1:2	0.996	0.0806 ± 0.0015	8.6 ± 0.2 d	***
		1:5	0.996	0.0187 ± 0.0003	37.1 ± 0.6 d	**			1:5	0.994	0.0629 ± 0.0014	11.0 ± 0.2 d	***
		1:10	0.996	0.0176 ± 0.0003	39.3 ± 0.7 d	***			1:10	0.986	0.0523 ± 0.0017	13.3 ± 0.4 d	***

Actual juice	90	1:0	0.990	0.0097 ± 0.0003	71.2 ± 2.2 min		Actual juice	90	1:0	0.993	0.0113 ± 0.0003	61.1 ± 1.7 min	
		1:1	0.998	0.0092 ± 0.0001	75.5 ± 1.1 min	ns			1:1	0.994	0.0109 ± 0.0003	63.7 ± 1.5 min	ns
		1:2	0.996	0.0089 ± 0.0002	77.8 ± 1.4 min	*			1:2	0.983	0.0104 ± 0.0004	66.7 ± 2.8 min	ns
		1:5	0.999	0.0080 ± 0.0001	86.3 ± 1.0 min	***			1:5	0.991	0.0099 ± 0.0003	69.9 ± 2.0 min	**
		1:10	0.999	0.0079 ± 0.0001	87.7 ± 0.9 min	***			1:10	0.997	0.0096 ± 0.0002	72.5 ± 1.2 min	***
	37	1:0	0.995	0.0850 ± 0.0017	8.2 ± 0.2 d			37	1:0	0.993	0.1258 ± 0.0032	5.5 ± 0.1 d	
		1:1	0.993	0.0801 ± 0.0019	8.7 ± 0.2 d	ns			1:1	0.998	0.1140 ± 0.0016	6.1 ± 0.1 d	**
		1:2	0.993	0.0782 ± 0.0018	8.9 ± 0.2 d	**			1:2	0.996	0.1063 ± 0.0019	6.5 ± 0.1 d	***
		1:5	0.989	0.0665 ± 0.0020	10.4 ± 0.3 d	***			1:5	0.997	0.0960 ± 0.0016	7.2 ± 0.1 d	***
		1:10	0.994	0.0623 ± 0.0014	11.1 ± 0.2 d	***			1:10	0.994	0.0827 ± 0.0018	8.4 ± 0.2 d	***

Values are mean ± SE. The rate constant *k, t*_1/2_ and their SE were obtained from nonlinear regression (first-order model, GraphPad Prism 11.0, extra sum-of-squares F test). Significance of difference in k between each treatment and the control was assessed by the extra sum-of-squares F test with Holm-Bonferroni correction; ns, not significant (*P* ≥ 0.05); * (*P* < 0.05); ** (*P* < 0.01); *** (*P* < 0.001).

Under conditions without AA, whether in the model or actual juice, and during either 90 °C heating or 37 °C storage, the degradation rate constant (*k*) of C3G generally decreased as the myricitrin ratio increased, with a corresponding extension of the half-life (t_1/2_). Under the harsh conditions with added AA, myricitrin still effectively counteracted oxidative stress, and higher concentrations (1:5, 1:10) provided better protection. The concentration-dependent protective effect of copigments on anthocyanins has also been reported for ferulic acid (Kanha et al., 2019). This characteristic appears uncertain for gallic acid. One study found that increasing levels of gallic acid from 0 to 320 mg/100 mL showed a consistent positive effect on anthocyanin retention in cranberry juice with added vitamin C. In contrast, another study demonstrated that higher concentrations of gallic acid (at anthocyanin-to-gallic acid molar ratios of 1:0, 1:1, 1:3, and 1:5) did not significantly enhance the C3G content in blueberry juice after 10 days of storage at 40 °C (L. Zhang et al., 2020). It seems that different food matrices, such as the presence of high levels of ascorbic acid, may influence the concentration dependence of the protective effect offered by copigments.

The concentration-dependent protective effect of myricitrin is directly related to the stoichiometry of the copigmentation complex formed with C3G. This is the macroscopic manifestation of the concentration-dependent spectral enhancement effects observed in Section 3.1. A higher concentration means more myricitrin molecules in the system are available to competitively stabilize C3G through copigmentation and potential antioxidant action. It is noteworthy that in the actual juice, the increase in half-life afforded by the exogenous addition of myricitrin may be relatively moderate because the matrix itself already provides considerable stability. This suggests that the “background effect” of the natural matrix needs to be considered in practical applications. This clear concentration-effect relationship provides direct scientific guidance for optimizing the addition level of myricitrin in actual production to achieve the best balance between cost, sensory properties, and stability.

### 3.4 Global nonlinear least squares (GNLS) analysis

A global nonlinear least squares (GNLS) model was fitted to all 1437 observations based on first-order kinetics, with log(*k*) parameterized as a linear function of fixed effects and separate residual variances for each of the eight experimental conditions. The model showed good fit (pseudo-*R*^2^ = 0.993, standardized residuals: mean ≈ 0, SD ≈ 0.94, range − 4.31 to 6.07).

In the full-sample model, main effects of copigment treatment, matrix, temperature, and AA were all significant (*P* < 0.001). All interactions up to the four-way level were significant; the four-way interaction (copigment treatment × matrix × temperature × AA) yielded *F*(9,1277) = 200.9, *P* < 0.001 (**Table S2**).

For the copigment type sub-model (fixed 1:5 ratio, *n* = 1006), the four-way interaction was also significant (*F*(6,894) = 202.1, *P* < 0.001). Dunnett comparisons (**Table S3**) revealed condition-dependent reversals: in model juice at 37 °C without AA, protocatechuic acid, ferulic acid, quercitrin, and rutin significantly accelerated degradation (*k* ratios 5.30–4.57, all *P* < 0.001), whereas in actual juice under the same conditions, these copigments showed significant protection (*k* ratios 0.80–0.91, *P* ≤ 0.005). At 90 °C, most copigments were ineffective in model juice, but rutin and quercitrin still significantly slowed degradation in Actual juice (*k* ratios 0.66–0.86, *P* < 0.001).

In the myricitrin concentration sub-model (*n* = 719), the main effect of concentration was significant (*F*(4,639) = 179.0, *P* < 0.001). Three three-way interactions reached significance: concentration × matrix × temperature (*F* = 3.17, *P* = 0.014), concentration × matrix × AA (*F* = 9.76, *P* < 0.001), and Concentration × temperature × AA (*F* = 3.45, *P* = 0.008). The four-way interaction was not included. Dunnett comparisons showed that *k* ratios generally decreased with increasing concentration (1:1 to 1:10), with strongest protection at 1:10 (e.g., model juice, 90 °C, with AA: *k* ratio from 0.88 to 0.57, all *P* < 0.001). However, in model juice at 37 °C without AA, none of the concentrations reached significance (*P* ≥ 0.091).

Overall, GNLS analysis demonstrates that the effects of copigments on C3G degradation are complexly modulated by matrix, temperature, and AA, with both copigment type and concentration exhibiting condition-dependent behaviors.

### 3.5 Effects of copigments on the color of Chinese bayberry juice

Color is the most direct indicator for evaluating the quality of anthocyanin-rich products. To investigate whether the color changes in Chinese bayberry juice are consistent with the findings on copigmentation and anthocyanin degradation, we measured the CIELab color parameters (L*, a*, b*) of the model Chinese bayberry juice after adding copigments (Table 4, Table 5). Additionally, we photographed the samples under identical conditions, a method widely used in studies on anthocyanin color (Gérard et al., 2019; Türkyılmaz et al., 2019). It can be observed that while the simulated colors can also reflect color differences, the actual photographs captured contain richer information, particularly allowing for the observation of differences in the hue of the Chinese bayberry juice.

**Table 4:** Color parameters (L*, a*, b*) of model Chinese bayberry juice containing cyanidin-3-O-glucoside (C3G; 0.16 mM) with different copigments (0.8 mM) after heating at 90 °C and storage at 37°C in the dark.

Temperature and time	Color	Copigment						
		Control	Protocatechuic acid	Gallic acid	Ferulic acid	Quercitrin	Myricitrin	Rutin
90 °C 0 min	L*	38.44 ± 0.31a	37.48 ± 0.36b	37.98 ± 0.40ab	37.38 ± 0.32b	35.14 ± 0.83c	33.07 ± 0.47d	34.24 ± 0.60e
	a*	30.09 ± 0.76c	30.12 ± 0.40c	29.72 ± 0.13c	30.00 ± 0.75c	33.27 ± 1.00b	35.15 ± 0.13a	34.46 ± 0.84a
	b*	12.25 ± 0.25b	13.66 ± 0.30a	12.04 ± 0.11b	11.04 ± 0.43c	7.84 ± 0.28e	8.36 ± 0.16d	7.18 ± 0.36f
	Color simulation							
	actual color							
90 °C 90 min	L*	43.95 ± 0.30a	43.73 ± 0.31ab	43.01 ± 0.37b	43.37 ± 0.11ab	40.67 ± 0.63c	37.37 ± 0.80d	40.56 ± 0.34c
	a*	17.63 ± 0.05e	18.49 ± 0.91de	20.10 ± 0.46c	19.02 ± 0.02 cd	21.98 ± 0.90b	27.09 ± 1.15a	21.92 ± 0.73b
	b*	7.74 ± 0.20 cd	8.14 ± 0.34bc	8.71 ± 0.19ab	8.88 ± 0.06a	6.36 ± 0.55e	7.04 ± 0.57de	7.08 ± 0.57de
	*ΔE*	14.35 ± 0.21a	14.32 ± 0.89a	11.35 ± 0.57bc	12.69 ± 0.89a	12.67 ± 1.01ab	9.25 ± 1.32c	14.05 ± 0.80a
	Color simulation							
	actual color							
37°C 0 d	L*	40.06 ± 0.46a	37.33 ± 0.22c	38.61 ± 0.35b	38.42 ± 0.37b	34.61 ± 0.16d	34.96 ± 0.18d	34.83 ± 0.78d
	a*	25.78 ± 0.79c	30.36 ± 0.50b	29.07 ± 0.62b	28.92 ± 0.06b	32.78 ± 0.70a	32.67 ± 0.79a	32.54 ± 1.40a
	b*	9.98 ± 0.28c	13.87 ± 0.48a	11.46 ± 0.57b	10.30 ± 0.09c	6.80 ± 0.15de	7.31 ± 0.62d	6.33 ± 0.26e
	Color simulation							
	actual color							
37°C 8 d	L*	40.98 ± 0.37a	40.13 ± 0.10bc	39.88 ± 0.51c	40.78 ± 0.27ab	35.97 ± 0.03d	36.25 ± 0.44d	35.50 ± 0.58d
	a*	24.72 ± 0.26c	25.46 ± 0.35b	25.88 ± 0.18b	23.28 ± 0.23d	30.71 ± 0.93a	30.97 ± 0.27a	30.37 ± 0.16a
	b*	9.37 ± 0.32a	9.96 ± 0.60a	9.67 ± 0.25a	8.48 ± 0.23b	6.67 ± 0.54 cd	7.01 ± 0.24c	6.04 ± 0.17d
	*ΔE*	1.56 ± 0.37	6.89 ± 0.27a	3.89 ± 0.40b	6.39 ± 0.17	2.54 ± 0.81c	2.17 ± 0.47c	2.33 ± 0.21c
	Color simulation							
	actual color							

Values are mean ± SD (*n* = 3). Different letters within the same row indicate significant differences (*P* < 0.05).

**Table 5:** Color parameters (L*, a*, b*) of model Chinese bayberry juice containing cyanidin-3-O-glucoside (C3G; 0.16 mM) as affected by different concentrations of myricitrin (molar ratios of C3G to myricitrin from 1:0 to 1:10) after heating at 90 °C and storage at 37 °C in the dark.

Temperature and time	Color	Molar ratio of C3G to myricitrin				
		1:0	1:1	1:2	1:5	1:10
90 °C 0 min	L*	38.44 ± 0.31a	36.62 ± 0.12b	35.52 ± 0.54c	33.07 ± 0.47d	30.53 ± 0.72e
	a*	30.09 ± 0.76d	32.19 ± 1.25c	32.60 ± 1.00c	35.15 ± 0.13b	37.90 ± 0.16a
	b*	12.25 ± 0.25a	11.53 ± 0.78a	10.41 ± 0.51b	8.36 ± 0.16c	6.79 ± 0.22d
	Color simulation					
	actual color					
90 °C 90 min	L*	43.95 ± 0.30a	41.97 ± 0.31b	39.99 ± 0.67c	37.37 ± 0.80d	35.69 ± 0.78e
	a*	17.63 ± 0.05d	21.30 ± 0.98c	23.67 ± 0.68b	27.09 ± 1.15a	28.67 ± 1.51a
	b*	7.74 ± 0.20a	8.10 ± 0.39a	7.81 ± 0.15a	7.04 ± 0.57b	5.92 ± 0.17c
	*ΔE*	14.35 ± 0.21a	12.61 ± 1.07ab	10.34 ± 0.32bc	9.25 ± 1.32c	10.61 ± 1.67bc
	Color simulation					
	actual color					
37°C 0 d	L*	40.06 ± 0.46a	38.36 ± 0.44b	37.53 ± 0.31c	34.96 ± 0.18d	32.83 ± 0.38e
	a*	25.78 ± 0.79d	28.28 ± 0.71c	28.97 ± 0.50c	32.67 ± 0.79b	35.09 ± 1.01a
	b*	9.98 ± 0.28a	9.21 ± 0.30b	8.35 ± 0.30c	7.31 ± 0.62d	6.14 ± 0.43e
	Color simulation					
	actual color					
37°C 8 d	L*	40.98 ± 0.37a	39.01 ± 0.21b	37.98 ± 0.45c	36.25 ± 0.44d	33.25 ± 0.22e
	a*	24.72 ± 0.26d	28.19 ± 0.36c	28.73 ± 0.74c	30.97 ± 0.27b	33.73 ± 0.20a
	b*	9.37 ± 0.32a	9.12 ± 0.37a	8.50 ± 0.37b	7.01 ± 0.24c	6.09 ± 0.17d
	*ΔE*	1.56 ± 0.37ab	0.80 ± 0.08b	0.83 ± 0.52b	2.17 ± 0.47a	1.44 ± 0.25ab
	Color simulation					
	actual color					

Values are mean ± SD (*n* = 3). Different letters within the same row indicate significant differences (*P* < 0.05).

By comparing the color changes in the model Chinese bayberry juice after adding the six copigments (Table 4), flavonoid glycosides (myricitrin, rutin, quercitrin) significantly (*p* < 0.05) improved and maintained the red color of the juice compared to phenolic acids. This is mainly reflected in a redder color at the beginning of heating or storage for the flavonoid glycoside groups, while a more pronounced yellow tint is observed in the phenolic acid groups. Furthermore, before and after heat treatment or storage, although all copigment-added groups effectively reduced lightness (L*), increased redness (a*), and decreased yellowness (b*), a trend consistent with previous findings (Lyu et al., 2025; Torres-Rochera et al., 2024; X. Wang, et al., 2024), the flavonoid glycoside groups retained a deeper color with a less yellow tint than the phenolic acid groups. For example, at 0 min of 90 °C heating, the myricitrin group had the lowest L*(33.07) and the highest a* (35.15). After 90 min of heating, its a* (27.09) remained significantly higher than that of the control group (17.63). It can be inferred that, in addition to protecting anthocyanins during heating and storage, leading to a higher residual anthocyanin content, the hyperchromic and bathochromic effects induced by myricitrin as a copigment also play an important role in improving the color.

The concentration effect of myricitrin further confirmed the concentration dependence of its action (Table 4). As its addition ratio increased from 1:0 to 1:10, the solution redness significantly intensified (a* increased continuously from 30.09 to 37.90), the color became purer (b* decreased continuously), and the color became darker (L* decreased continuously). This is consistent with the pattern observed in a model red wine system (whose anthocyanin is malvidin-3-O-glucoside) (B. Zhang et al., 2017). Even after the harsh treatment of 90 °C heating for 90 min, the high-concentration myricitrin treatment groups still maintained the best color.

The decrease in L* value and increase in a* value are the macroscopic manifestations of copigmentation, corresponding to the previously observed hyperchromic and bathochromic spectral phenomena. The color data visually demonstrate that effective copigments like myricitrin and rutin can not only kinetically slow down the chemical degradation of C3G but also directly enhance the visual quality of the product on a macroscopic level. The concentration-dependent color protection by myricitrin also provides strong visual evidence for its concentration-dependent stability effect. However, it is important to note that the color performance of the Chinese bayberry juice was not completely consistent with the anthocyanin content. For example, after 8 days of storage at 37 °C, although the C3G content in the quercitrin and rutin groups was significantly lower than the control (*p* < 0.05), the color performance of the former two was still better than the control **(**Table 5**)**. Another example: after 8 days of storage at 37 °C, although the C3G content in the protocatechuic acid and ferulic acid groups was significantly lower than the control (*p* < 0.05), the color performance of the three was very similar. This suggests that in certain matrices, the color changes induced by copigmentation have a greater impact on the overall color performance of the system than the inherent color of the anthocyanins themselves.

To further quantify the relationship between color change and anthocyanin degradation, total color difference (*ΔE*) was calculated for all treatments (Table 4, Table 5). In general, *ΔE* increased after heating or storage, reflecting cumulative color deterioration. Treatments with flavonoid glycoside copigments (myricitrin, rutin, quercitrin) exhibited lower *ΔE* values compared to phenolic acid groups and the control, consistent with their higher C3G retention. For example, after 90 min at 90 °C, the myricitrin group showed *ΔE* = 9.25, substantially lower than the control (*ΔE* = 14.35). Pearson correlation analysis (**Fig. S1**) was performed on two representative conditions (model juice, 90 °C, no AA and model juice, 37 °C, no AA). C3G retention was significantly negatively correlated with ΔE (90 °C: *r* = −0.728, *P* < 0.05; 37 °C: *r* = −0.749, *P* < 0.05) and significantly positively correlated with a* (90 °C: *r* = 0.951, *P* < 0.01; 37 °C: *r* = 0.938, *P* < 0.01). These results statistically confirm the consistency between C3G degradation and color deterioration.

## 4 Conclusions

This study systematically investigated the condition-dependent effects of six phenolic copigments on the stability of cyanidin-3-O-glucoside (C3G) in both model and actual Chinese bayberry juice systems by combining visible absorption spectroscopy, degradation kinetics, and color analysis. The findings reveal that flavonoid glycoside copigments (rutin, myricitrin, quercitrin) exhibit significant bathochromic shifts and hyperchromic effects, consistent with π-π stacking interactions with C3G facilitated by their planar conjugated structures. Consequently, they effectively delay C3G degradation under most conditions, including during 90 °C heat treatment, and improve the red color of the juice. However, this protective effect is highly condition-dependent. During long-term storage at 37 °C in the simplified model juice, some flavonoid glycosides instead accelerated degradation. In contrast, the complex matrix of the actual Chinese bayberry juice effectively maintained the functionality of the copigments, highlighting the crucial influence of the food matrix composition on copigmentation in real food systems. This study offers insights that may inform the regulation of color and stability of Chinese bayberry juice: the use of flavonoid glycoside copigments such as myricitrin is recommended, and retaining the natural juice matrix during processing may help support copigment functionality.

From a practical perspective, the findings of this study provide preliminary guidance for the selection and dosage optimization of copigments in Chinese bayberry juice processing. Flavonoid glycoside copigments, particularly myricitrin, exhibited optimal color protection at molar ratios of 1:5 to 1:10. However, several factors need to be considered before industrial application: (1) the impact on sensory attributes such as flavor and mouthfeel requires consumer acceptance testing; (2) although the selected copigments are naturally occurring flavonoids aligned with clean-label trends, their regulatory status varies by jurisdiction; (3) the cost-effectiveness of large-scale extraction and purification warrants further assessment. Bridging the gap between laboratory findings and industrial implementation will require additional translational studies.

Nevertheless, several limitations of this study should be acknowledged. First, only six phenolic copigments were evaluated, representing a limited subset of the diverse phenolic pool present in plant-based foods. Second, all experiments were conducted at a fixed pH of 3.3 (typical of Chinese bayberry juice) and in the absence of light; the effects of pH variation and light exposure—both of which are known to influence anthocyanin stability and copigmentation—remain unexplored. Third, while spectroscopic and kinetic evidence suggests the involvement of π-π stacking, direct molecular-level characterization (e.g., NMR or computational modeling) was not performed. Fourth, pH was not monitored continuously during the heating and storage periods. Future studies should expand the copigment library to include other abundant dietary phenolics (e.g., hydroxycinnamic acids, flavanols) and investigate their synergistic or antagonistic interactions. Systematic evaluation across a broader pH range and under light/dark cycles would provide more comprehensive guidance for industrial processing and storage. Additionally, the mechanism by which certain copigments transition from protective to degradation-enhancing agents under specific storage conditions warrants targeted investigation, ideally combining kinetic profiling with identification of degradation intermediates and measurement of oxidative markers. Such work will facilitate the rational design of copigment strategies tailored to specific food matrices and processing conditions.

## CRediT authorship contribution statement

**Zhengwei Zhang:** Writing – original draft, Software, Methodology, Formal analysis, Data curation. **Xiaodan Chen:** Methodology, Data curation. **Jinning Yin:** Data curation. **Luelue Huang:** Writing – review & editing, Supervision, Resources.

## Declaration of competing interest

The authors declare that they have no known competing financial interests or personal relationships that could have appeared to influence the work reported in this paper.

## Data Availability

Data will be made available on request.
