# Supplementary material for: Condition-dependent effects of phenolic Copigments on anthocyanin stability in Chinese bayberry juice: An HPLC-degradation kinetic study
Source: Food Chem X. 2026 Aug 27;39:104368. doi: 10.1016/j.fochx.2026.104368 (PMC13551933; doi:10.1016/j.fochx.2026.104368)
Supplement: Supplementary file 1 [file mmc1.docx]

Table S1. Basic composition of the Chinese bayberry juice matrix.

| Composition | Content |
| --- | --- |
| pH | 3.25 ± 0.01 |
| Total sugar (%) | 9.45 ± 0.42 |
| Titratable acidity (TA, %) | 0.96 ± 0.03 |
| Soluble solids content (SSC, %) | 8.9 ± 0.2 |
| Cyanidin-3-O-glucoside (mg/L) | 77.57 ± 0.26 |
| Total phenols content (mg GAE/L) | 362.87 ± 11.15 |
| Quercetin (mg/L) | 0.40 ± 0.00 |
| Kaempferol (mg/L) | 0.01 ± 0.01 |
| Gallic acid (mg/L) | 0.06 ± 0.00 |
| *p*-Hydroxybenzoic acid (mg/L) | 1.13 ± 0.19 |
| *p*-Coumaric acid (mg/L) | 0.24 ± 0.04 |
| Ferulic acid (mg/L) | 0.50 ± 0.07 |
| (-)-Epicatechin (mg/L) | 0.03 ± 0.00 |
| Hyperoside (mg/L) | 0.26 ± 0.01 |
| Myricitrin (mg/L) | 17.89 ± 0.49 |
| Quercitrin (mg/L) | 5.73 ± 0.17 |
| Protocatechuic acid (mg/L) | 0.82 ± 0.11 |
| Myricetin (mg/L) | 5.14 ± 0.62 |

AA was not measured. For reference, Yu, Lin, Zhan, He, and Zhu (2013) reported 21.21 ± 1.24 mg/100 mL AA in Chinese bayberry juice.

Table S2. ANOVA results for the global nonlinear models.

| Model | Effect | Numerator df | Denominator df | F | P |
| --- | --- | --- | --- | --- | --- |
| Full-Sample Combined Treatment Model | Copigment Treatment (Type + Concentration) | 9 | 1277 | 105.798 | <0.001 |
| Full-Sample Combined Treatment Model | Matrix | 1 | 1277 | 3072.247 | <0.001 |
| Full-Sample Combined Treatment Model | Temperature | 1 | 1277 | 437294.020 | <0.001 |
| Full-Sample Combined Treatment Model | AA | 1 | 1277 | 1170.103 | <0.001 |
| Full-Sample Combined Treatment Model | Copigment Treatment (Type + Concentration) × Matrix | 9 | 1277 | 27.371 | <0.001 |
| Full-Sample Combined Treatment Model | Copigment Treatment (Type + Concentration) × Temperature | 9 | 1277 | 8.409 | <0.001 |
| Full-Sample Combined Treatment Model | Matrix × Temperature | 1 | 1277 | 1371.728 | <0.001 |
| Full-Sample Combined Treatment Model | Copigment Treatment (Type + Concentration) × AA | 9 | 1277 | 231.257 | <0.001 |
| Full-Sample Combined Treatment Model | Matrix × AA | 1 | 1277 | 11.125 | <0.001 |
| Full-Sample Combined Treatment Model | Temperature × AA | 1 | 1277 | 107.809 | <0.001 |
| Full-Sample Combined Treatment Model | Copigment Treatment (Type + Concentration) × Matrix × Temperature | 9 | 1277 | 5.236 | <0.001 |
| Full-Sample Combined Treatment Model | Copigment Treatment (Type + Concentration) × Matrix × AA | 9 | 1277 | 225.570 | <0.001 |
| Full-Sample Combined Treatment Model | Copigment Treatment (Type + Concentration) × Temperature × AA | 9 | 1277 | 258.069 | <0.001 |
| Full-Sample Combined Treatment Model | Matrix × Temperature × AA | 1 | 1277 | 239.813 | <0.001 |
| Full-Sample Combined Treatment Model | Copigment Treatment (Type + Concentration) × Matrix × Temperature × AA | 9 | 1277 | 200.866 | <0.001 |
| Copigment Type Model (Common 1:5 Ratio) | Copigment Type | 6 | 894 | 98.424 | <0.001 |
| Copigment Type Model (Common 1:5 Ratio) | Matrix | 1 | 894 | 1712.542 | <0.001 |
| Copigment Type Model (Common 1:5 Ratio) | Temperature | 1 | 894 | 299232.200 | <0.001 |
| Copigment Type Model (Common 1:5 Ratio) | AA | 1 | 894 | 206.900 | <0.001 |
| Copigment Type Model (Common 1:5 Ratio) | Copigment Type × Matrix | 6 | 894 | 26.909 | <0.001 |
| Copigment Type Model (Common 1:5 Ratio) | Copigment Type × Temperature | 6 | 894 | 11.776 | <0.001 |
| Copigment Type Model (Common 1:5 Ratio) | Matrix × Temperature | 1 | 894 | 947.142 | <0.001 |
| Copigment Type Model (Common 1:5 Ratio) | Copigment Type × AA | 6 | 894 | 232.582 | <0.001 |
| Copigment Type Model (Common 1:5 Ratio) | Matrix × AA | 1 | 894 | 131.729 | <0.001 |
| Copigment Type Model (Common 1:5 Ratio) | Temperature × AA | 1 | 894 | 546.744 | <0.001 |
| Copigment Type Model (Common 1:5 Ratio) | Copigment Type × Matrix × Temperature | 6 | 894 | 6.777 | <0.001 |
| Copigment Type Model (Common 1:5 Ratio) | Copigment Type × Matrix × AA | 6 | 894 | 224.398 | <0.001 |
| Copigment Type Model (Common 1:5 Ratio) | Copigment Type × Temperature × AA | 6 | 894 | 265.788 | <0.001 |
| Copigment Type Model (Common 1:5 Ratio) | Matrix × Temperature × AA | 1 | 894 | 690.250 | <0.001 |
| Copigment Type Model (Common 1:5 Ratio) | Copigment Type × Matrix × Temperature × AA | 6 | 894 | 202.116 | <0.001 |
| Myricitrin Concentration Model | Myricitrin Concentration | 4 | 639 | 178.975 | <0.001 |
| Myricitrin Concentration Model | Matrix | 1 | 639 | 3146.703 | <0.001 |
| Myricitrin Concentration Model | Temperature | 1 | 639 | 317593.125 | <0.001 |
| Myricitrin Concentration Model | AA | 1 | 639 | 3987.735 | <0.001 |
| Myricitrin Concentration Model | Myricitrin Concentration × Matrix | 4 | 639 | 9.597 | <0.001 |
| Myricitrin Concentration Model | Myricitrin Concentration × Temperature | 4 | 639 | 1.880 | 0.112 |
| Myricitrin Concentration Model | Matrix × Temperature | 1 | 639 | 1013.325 | <0.001 |
| Myricitrin Concentration Model | Myricitrin Concentration × AA | 4 | 639 | 9.902 | <0.001 |
| Myricitrin Concentration Model | Matrix × AA | 1 | 639 | 1295.613 | <0.001 |
| Myricitrin Concentration Model | Temperature × AA | 1 | 639 | 683.239 | <0.001 |
| Myricitrin Concentration Model | Myricitrin Concentration × Matrix × Temperature | 4 | 639 | 3.166 | 0.014 |
| Myricitrin Concentration Model | Myricitrin Concentration × Matrix × AA | 4 | 639 | 9.762 | <0.001 |
| Myricitrin Concentration Model | Myricitrin Concentration × Temperature × AA | 4 | 639 | 3.451 | 0.008 |
| Myricitrin Concentration Model | Matrix × Temperature × AA | 1 | 639 | 304.193 | <0.001 |
| Myricitrin Concentration Model | Myricitrin Concentration × Matrix × Temperature × AA | 4 | 639 | 2.261 | 0.061 |

Table S3. Dunnett post-hoc comparisons of degradation rate constants between each treatment and its respective control in the GNLS models.

| Matrix | T (°C) | AA | Comparison Type | Copigment | Ratio | Adjusted *k* (Treatment) | Adjusted *k* (Control) | *k* Difference (Treatment - Control) | *k* Ratio (Treatment / Control) | Dunnett-Adjusted 95% CI Lower Bound | Dunnett-Adjusted 95% CI Upper Bound | *t* | Raw *P* | Dunnett-Adjusted *P* |
| --- | --- | --- | --- | --- | --- | --- | --- | --- | --- | --- | --- | --- | --- | --- |
| Model | 37 | without | Myricitrin Concentration vs. Control | Myricitrin | 1:1 | 0.001 | 0.001 | 0.000 | 0.834 | 0.657 | 1.058 | -1.991 | 0.047 | 0.206 |
| Model | 90 | with | Myricitrin Concentration vs. Control | Myricitrin | 1:1 | 0.676 | 0.772 | -0.096 | 0.875 | 0.828 | 0.926 | -6.420 | <0.001 | <0.001 |
| Actual | 90 | without | Myricitrin Concentration vs. Control | Myricitrin | 1:1 | 0.551 | 0.584 | -0.033 | 0.943 | 0.864 | 1.030 | -1.792 | 0.073 | 0.377 |
| Actual | 90 | with | Myricitrin Concentration vs. Control | Myricitrin | 1:1 | 0.653 | 0.680 | -0.028 | 0.959 | 0.888 | 1.036 | -1.459 | 0.145 | 0.626 |
| Model | 90 | without | Myricitrin Concentration vs. Control | Myricitrin | 1:1 | 0.379 | 0.415 | -0.036 | 0.913 | 0.768 | 1.085 | -1.426 | 0.154 | 0.644 |
| Actual | 37 | with | Myricitrin Concentration vs. Control | Myricitrin | 1:1 | 0.005 | 0.005 | 0.000 | 0.906 | 0.839 | 0.979 | -3.477 | <0.001 | 0.004 |
| Model | 37 | with | Myricitrin Concentration vs. Control | Myricitrin | 1:1 | 0.004 | 0.004 | 0.000 | 0.961 | 0.878 | 1.051 | -1.228 | 0.220 | 0.859 |
| Actual | 37 | without | Myricitrin Concentration vs. Control | Myricitrin | 1:1 | 0.003 | 0.004 | 0.000 | 0.943 | 0.877 | 1.013 | -2.208 | 0.027 | 0.170 |
| Actual | 90 | with | Myricitrin Concentration vs. Control | Myricitrin | 1:2 | 0.624 | 0.680 | -0.057 | 0.917 | 0.848 | 0.991 | -3.033 | 0.002 | 0.019 |
| Actual | 90 | without | Myricitrin Concentration vs. Control | Myricitrin | 1:2 | 0.534 | 0.584 | -0.050 | 0.915 | 0.837 | 1.000 | -2.712 | 0.007 | 0.048 |
| Actual | 37 | without | Myricitrin Concentration vs. Control | Myricitrin | 1:2 | 0.003 | 0.004 | 0.000 | 0.920 | 0.856 | 0.989 | -3.115 | 0.002 | 0.015 |
| Actual | 37 | with | Myricitrin Concentration vs. Control | Myricitrin | 1:2 | 0.004 | 0.005 | -0.001 | 0.845 | 0.780 | 0.914 | -5.796 | <0.001 | <0.001 |
| Model | 37 | without | Myricitrin Concentration vs. Control | Myricitrin | 1:2 | 0.001 | 0.001 | 0.000 | 0.784 | 0.614 | 1.000 | -2.602 | 0.009 | 0.050 |
| Model | 90 | with | Myricitrin Concentration vs. Control | Myricitrin | 1:2 | 0.609 | 0.772 | -0.164 | 0.788 | 0.744 | 0.835 | -11.248 | <0.001 | <0.001 |
| Model | 90 | without | Myricitrin Concentration vs. Control | Myricitrin | 1:2 | 0.354 | 0.415 | -0.061 | 0.853 | 0.716 | 1.017 | -2.435 | 0.015 | 0.100 |
| Model juice | 37 | with | Myricitrin Concentration vs. Control | Myricitrin | 1:2 | 0.003 | 0.004 | -0.001 | 0.839 | 0.763 | 0.921 | -5.145 | <0.001 | <0.001 |
| Actual juice | 90 | without | Myricitrin Concentration vs. Control | Myricitrin | 1:5 | 0.482 | 0.584 | -0.102 | 0.826 | 0.754 | 0.904 | -5.718 | <0.001 | <0.001 |
| Actual juice | 90 | with | Myricitrin Concentration vs. Control | Myricitrin | 1:5 | 0.595 | 0.680 | -0.085 | 0.875 | 0.810 | 0.946 | -4.642 | <0.001 | <0.001 |
| Actual juice | 37 | with | Myricitrin Concentration vs. Control | Myricitrin | 1:5 | 0.004 | 0.005 | -0.001 | 0.763 | 0.704 | 0.827 | -9.140 | <0.001 | <0.001 |
| Actual juice | 37 | without | Myricitrin Concentration vs. Control | Myricitrin | 1:5 | 0.003 | 0.004 | -0.001 | 0.783 | 0.725 | 0.845 | -8.650 | <0.001 | <0.001 |
| Model juice | 90 | with | Myricitrin Concentration vs. Control | Myricitrin | 1:5 | 0.514 | 0.772 | -0.258 | 0.666 | 0.627 | 0.707 | -18.456 | <0.001 | <0.001 |
| Model juice | 37 | with | Myricitrin Concentration vs. Control | Myricitrin | 1:5 | 0.003 | 0.004 | -0.001 | 0.654 | 0.590 | 0.725 | -11.386 | <0.001 | <0.001 |
| Model juice | 90 | without | Myricitrin Concentration vs. Control | Myricitrin | 1:5 | 0.317 | 0.415 | -0.098 | 0.765 | 0.637 | 0.918 | -3.972 | <0.001 | <0.001 |
| Model juice | 37 | without | Myricitrin Concentration vs. Control | Myricitrin | 1:5 | 0.001 | 0.001 | 0.000 | 0.807 | 0.637 | 1.022 | -2.366 | 0.018 | 0.091 |
| Actual juice | 90 | without | Myricitrin Concentration vs. Control | Myricitrin | 1:10 | 0.474 | 0.584 | -0.110 | 0.812 | 0.742 | 0.889 | -6.248 | <0.001 | <0.001 |
| Actual juice | 37 | with | Myricitrin Concentration vs. Control | Myricitrin | 1:10 | 0.003 | 0.005 | -0.002 | 0.657 | 0.604 | 0.715 | -13.524 | <0.001 | <0.001 |
| Model juice | 90 | with | Myricitrin Concentration vs. Control | Myricitrin | 1:10 | 0.441 | 0.772 | -0.332 | 0.571 | 0.536 | 0.608 | -24.102 | <0.001 | <0.001 |
| Actual juice | 37 | without | Myricitrin Concentration vs. Control | Myricitrin | 1:10 | 0.003 | 0.004 | -0.001 | 0.733 | 0.679 | 0.792 | -10.970 | <0.001 | <0.001 |
| Actual juice | 90 | with | Myricitrin Concentration vs. Control | Myricitrin | 1:10 | 0.574 | 0.680 | -0.107 | 0.843 | 0.779 | 0.912 | -5.879 | <0.001 | <0.001 |
| Model juice | 90 | without | Myricitrin Concentration vs. Control | Myricitrin | 1:10 | 0.291 | 0.415 | -0.123 | 0.703 | 0.575 | 0.859 | -4.737 | <0.001 | <0.001 |
| Model juice | 37 | with | Myricitrin Concentration vs. Control | Myricitrin | 1:10 | 0.002 | 0.004 | -0.002 | 0.544 | 0.487 | 0.607 | -15.294 | <0.001 | <0.001 |
| Model juice | 37 | without | Myricitrin Concentration vs. Control | Myricitrin | 1:10 | 0.001 | 0.001 | 0.000 | 0.762 | 0.599 | 0.971 | -2.928 | 0.003 | 0.020 |
| Model juice | 90 | with | Copigment Type vs. Control | Gallic acid | 1:5 | 0.579 | 0.772 | -0.194 | 0.749 | 0.706 | 0.795 | -13.267 | <0.001 | <0.001 |
| Actual juice | 90 | without | Copigment Type vs. Control | Gallic acid | 1:5 | 0.529 | 0.584 | -0.055 | 0.905 | 0.829 | 0.988 | -3.080 | 0.002 | 0.016 |
| Model juice | 37 | without | Copigment Type vs. Control | Gallic acid | 1:5 | 0.001 | 0.001 | 0.000 | 0.978 | 0.785 | 1.220 | -0.259 | 0.796 | 1.000 |
| Model juice | 90 | without | Copigment Type vs. Control | Gallic acid | 1:5 | 0.393 | 0.415 | -0.022 | 0.948 | 0.795 | 1.130 | -0.823 | 0.411 | 0.973 |
| Actual juice | 37 | with | Copigment Type vs. Control | Gallic acid | 1:5 | 0.004 | 0.005 | -0.001 | 0.785 | 0.722 | 0.853 | -7.915 | <0.001 | <0.001 |
| Actual juice | 90 | with | Copigment Type vs. Control | Gallic acid | 1:5 | 0.616 | 0.680 | -0.064 | 0.906 | 0.835 | 0.982 | -3.306 | <0.001 | 0.008 |
| Model juice | 37 | with | Copigment Type vs. Control | Gallic acid | 1:5 | 0.002 | 0.004 | -0.002 | 0.584 | 0.523 | 0.652 | -13.435 | <0.001 | <0.001 |
| Actual juice | 37 | without | Copigment Type vs. Control | Gallic acid | 1:5 | 0.003 | 0.004 | 0.000 | 0.899 | 0.836 | 0.968 | -3.886 | <0.001 | <0.001 |
| Actual juice | 90 | with | Copigment Type vs. Control | Ferulic acid | 1:5 | 0.669 | 0.680 | -0.012 | 0.983 | 0.907 | 1.066 | -0.577 | 0.564 | 0.998 |
| Actual juice | 90 | without | Copigment Type vs. Control | Ferulic acid | 1:5 | 0.542 | 0.584 | -0.042 | 0.929 | 0.850 | 1.014 | -2.267 | 0.024 | 0.147 |
| Actual juice | 37 | with | Copigment Type vs. Control | Ferulic acid | 1:5 | 0.004 | 0.005 | -0.001 | 0.844 | 0.778 | 0.916 | -5.637 | <0.001 | <0.001 |
| Model juice | 37 | without | Copigment Type vs. Control | Ferulic acid | 1:5 | 0.005 | 0.001 | 0.004 | 5.332 | 4.518 | 6.293 | 26.320 | <0.001 | <0.001 |
| Model juice | 90 | with | Copigment Type vs. Control | Ferulic acid | 1:5 | 0.746 | 0.772 | -0.026 | 0.966 | 0.914 | 1.022 | -1.664 | 0.096 | 0.473 |
| Actual juice | 37 | without | Copigment Type vs. Control | Ferulic acid | 1:5 | 0.004 | 0.004 | 0.000 | 1.035 | 0.964 | 1.112 | 1.312 | 0.190 | 0.735 |
| Model juice | 90 | without | Copigment Type vs. Control | Ferulic acid | 1:5 | 0.440 | 0.415 | 0.025 | 1.061 | 0.900 | 1.252 | 0.975 | 0.330 | 0.928 |
| Model juice | 37 | with | Copigment Type vs. Control | Ferulic acid | 1:5 | 0.001 | 0.004 | -0.003 | 0.337 | 0.289 | 0.393 | -19.384 | <0.001 | <0.001 |
| Actual juice | 90 | with | Copigment Type vs. Control | Quercitrin | 1:5 | 0.610 | 0.680 | -0.070 | 0.897 | 0.827 | 0.973 | -3.628 | <0.001 | 0.003 |
| Actual juice | 37 | with | Copigment Type vs. Control | Quercitrin | 1:5 | 0.004 | 0.005 | -0.001 | 0.744 | 0.682 | 0.811 | -9.364 | <0.001 | <0.001 |
| Actual juice | 90 | without | Copigment Type vs. Control | Quercitrin | 1:5 | 0.500 | 0.584 | -0.084 | 0.856 | 0.784 | 0.935 | -4.745 | <0.001 | <0.001 |
| Actual juice | 37 | without | Copigment Type vs. Control | Quercitrin | 1:5 | 0.003 | 0.004 | -0.001 | 0.835 | 0.774 | 0.900 | -6.506 | <0.001 | <0.001 |
| Model juice | 90 | with | Copigment Type vs. Control | Quercitrin | 1:5 | 0.634 | 0.772 | -0.138 | 0.821 | 0.776 | 0.869 | -9.394 | <0.001 | <0.001 |
| Model juice | 90 | without | Copigment Type vs. Control | Quercitrin | 1:5 | 0.390 | 0.415 | -0.024 | 0.941 | 0.792 | 1.118 | -0.950 | 0.342 | 0.937 |
| Model juice | 37 | without | Copigment Type vs. Control | Quercitrin | 1:5 | 0.004 | 0.001 | 0.003 | 3.718 | 3.142 | 4.399 | 20.328 | <0.001 | <0.001 |
| Model juice | 37 | with | Copigment Type vs. Control | Quercitrin | 1:5 | 0.001 | 0.004 | -0.003 | 0.171 | 0.132 | 0.222 | -18.530 | <0.001 | <0.001 |
| Actual juice | 90 | without | Copigment Type vs. Control | Protocatechuic acid | 1:5 | 0.527 | 0.584 | -0.057 | 0.902 | 0.825 | 0.986 | -3.132 | 0.002 | 0.014 |
| Actual juice | 37 | with | Copigment Type vs. Control | Protocatechuic acid | 1:5 | 0.004 | 0.005 | -0.001 | 0.819 | 0.754 | 0.889 | -6.588 | <0.001 | <0.001 |
| Actual juice | 37 | without | Copigment Type vs. Control | Protocatechuic acid | 1:5 | 0.003 | 0.004 | 0.000 | 0.909 | 0.843 | 0.979 | -3.458 | <0.001 | 0.005 |
| Actual juice | 90 | with | Copigment Type vs. Control | Protocatechuic acid | 1:5 | 0.648 | 0.680 | -0.032 | 0.953 | 0.877 | 1.036 | -1.568 | 0.117 | 0.543 |
| Model juice | 90 | without | Copigment Type vs. Control | Protocatechuic acid | 1:5 | 0.427 | 0.415 | 0.012 | 1.030 | 0.872 | 1.216 | 0.478 | 0.632 | 0.999 |
| Model juice | 90 | with | Copigment Type vs. Control | Protocatechuic acid | 1:5 | 0.731 | 0.772 | -0.042 | 0.946 | 0.894 | 1.001 | -2.655 | 0.008 | 0.057 |
| Model juice | 37 | with | Copigment Type vs. Control | Protocatechuic acid | 1:5 | 0.001 | 0.004 | -0.003 | 0.198 | 0.156 | 0.253 | -18.237 | <0.001 | <0.001 |
| Model juice | 37 | without | Copigment Type vs. Control | Protocatechuic acid | 1:5 | 0.005 | 0.001 | 0.004 | 5.299 | 4.489 | 6.256 | 26.171 | <0.001 | <0.001 |
| Model juice | 90 | with | Copigment Type vs. Control | Rutin | 1:5 | 0.664 | 0.772 | -0.108 | 0.860 | 0.813 | 0.910 | -7.212 | <0.001 | <0.001 |
| Actual juice | 90 | without | Copigment Type vs. Control | Rutin | 1:5 | 0.387 | 0.584 | -0.197 | 0.663 | 0.601 | 0.732 | -11.229 | <0.001 | <0.001 |
| Model juice | 90 | without | Copigment Type vs. Control | Rutin | 1:5 | 0.381 | 0.415 | -0.034 | 0.919 | 0.775 | 1.090 | -1.340 | 0.181 | 0.710 |
| Actual juice | 37 | with | Copigment Type vs. Control | Rutin | 1:5 | 0.003 | 0.005 | -0.002 | 0.659 | 0.603 | 0.721 | -12.686 | <0.001 | <0.001 |
| Model juice | 37 | without | Copigment Type vs. Control | Rutin | 1:5 | 0.004 | 0.001 | 0.003 | 4.566 | 3.866 | 5.394 | 23.750 | <0.001 | <0.001 |
| Model juice | 37 | with | Copigment Type vs. Control | Rutin | 1:5 | 0.001 | 0.004 | -0.003 | 0.166 | 0.127 | 0.217 | -18.407 | <0.001 | <0.001 |
| Actual juice | 37 | without | Copigment Type vs. Control | Rutin | 1:5 | 0.003 | 0.004 | -0.001 | 0.802 | 0.742 | 0.865 | -7.795 | <0.001 | <0.001 |
| Actual juice | 90 | with | Copigment Type vs. Control | Rutin | 1:5 | 0.601 | 0.680 | -0.079 | 0.883 | 0.812 | 0.960 | -4.009 | <0.001 | <0.001 |

The adjustment method is Dunnett's single-step procedure (multiplicity adjustment within the same experimental condition). AA means ascorbic acid.

|  |  |
| --- | --- |
|  |  |

Figure S1. Scatter plots of C3G retention versus color parameters (Pearson correlation) in two representative treatment groups. (A, B) Model, 90°C, no AA; (C, D) Model, 37°C, no AA.

| Control |  |
| --- | --- |
| Protocatechuic acid |  |
| Gallic acid |  |
| Ferulic acid |  |
| Quercitrin |  |
| Myricitrin |  |
| Rutin |  |

Figure S2. Representative HPLC chromatograms comparing the initial response of cyanidin-3-O-glucoside (C3G) in the presence of different copigments in model Chinese bayberry juice. Samples were analyzed at t = 0 min from the model juice system (0.16 mM C3G, pH 3.3) without ascorbic acid (AA), prior to heating at 90°C. Chromatograms are shown for the control (no copigment) and samples with each of the six copigments (protocatechuic acid, gallic acid, ferulic acid, quercitrin, myricitrin, and rutin) at a C3G-to-copigment molar ratio of 1:5.

| 1:0 |  |
| --- | --- |
| 1:1 |  |
| 1:2 |  |
| 1:5 |  |
| 1:10 |  |

Figure S3. Representative HPLC chromatograms comparing the initial response of cyanidin-3-O-glucoside (C3G) in the presence of myricitrin in model Chinese bayberry juice. Samples were analyzed at t = 0 min from the model juice system (0.16 mM C3G, pH 3.3) without ascorbic acid (AA), prior to heating at 90°C. Chromatograms are shown for the control (no copigment) and samples with myricitrin at C3G-to-copigment molar ratios of 1:1, 1:2, 1:5, and 1:10.
